# Reproducibility of late gadolinium enhancement quantification techniques in ischemic and non-ischemic heart diseases (ReLate study)

**DOI:** 10.3389/fcvm.2025.1621292

**Published:** 2025-08-01

**Authors:** Jonathan Gavrysh, Philine Reisdorf, Thomas Hadler, Thomas Mayr, Clemens Ammann, Jan Gröschel, Johanna Kuhnt, Florian von Knobelsdorff-Brenkenhoff, Fabian Muehlberg, Carsten Schwenke, Maximilian Fenski, Jeanette Schulz-Menger

**Affiliations:** ^1^ Working Group on CMR, Experimental and Clinical Research Center, a cooperation between the Max Delbrück Center for Molecular Medicine in the Helmholtz Association and Charité—Universitätsmedizin Berlin, Berlin, Germany; ^2^ Charité—Universitätsmedizin Berlin, corporate member of Freie Universität Berlin and Humboldt-Universität zu Berlin, Berlin, Germany; ^3^ DZHK (German Centre for Cardiovascular Research), partner site Berlin, Berlin, Germany; ^4^ Department of Cardiology and Nephrology, HELIOS Hospital Berlin-Buch, Berlin, Germany; ^5^ Department of Cardiology, Angiology and Intensive Care Medicine, Deutsches Herzzentrum der Charité, Berlin, Germany; ^6^ KIZ—Kardiologie im Zentrum and Ludwig-Maximilians-University Munich, Munich, Germany; ^7^ MSB Medical School Berlin, Berlin, Germany; ^8^ SCO:SSiS Statistical Consulting, Minden, Germany

**Keywords:** late gadolinium enhancement, myocardial scar, fibrosis, ischemic heart disease, inflammatory heart disease, hypertrophic cardiomyopathy, cardiovascular magnetic resonance, quantification technique

## Abstract

**Background:**

Late gadolinium enhancement (LGE) assessed by cardiovascular magnetic resonance (CMR) is an established metric for risk stratification and therapeutic guidance. However, consensus on the optimal technique for quantifying left ventricular (LV) LGE extent remains lacking. This study aimed to identify the most reliable method for quantifying LGE in chronic myocardial infarction (CMI), hypertrophic cardiomyopathy (HCM), and inflammatory heart disease (IHD).

**Methods:**

A retrospective analysis was conducted on 285 prospectively enrolled patients (CMI: *n* = 98; HCM: *n* = 91; IHD: *n* = 96). LV LGE images in short-axis orientation were analyzed twice by the same reader. The most reliable LGE quantification technique was defined as the one achieving the highest intra-observer reproducibility. A two-step study design was implemented: in the pilot phase (*n* = 90), three quantification methods were compared: full width at half maximum (FWHM), signal threshold vs. reference mean using 2–6 standard deviations (n-SD), and manual thresholding. Techniques demonstrating the lowest variability were then applied in a validation cohort (*n* = 195). A mixed model for repeated measures was used to estimate mean differences. Equivalence was confirmed if the 95% confidence interval (CI) for the mean difference remained within predefined margins.

**Results:**

In CMI, FWHM demonstrated the highest reproducibility, with a mean difference of 0.47% (95% CI: −0.40 to 1.35). In HCM, both the 5-SD and 6-SD techniques showed the highest reproducibility, with mean differences of 0.06% (95% CI: −1.28 to 1.39) and −0.16% (95% CI: −1.50 to 1.17), respectively. In IHD, the 5-SD and 6-SD techniques achieved the highest reproducibility, with mean differences of −0.72% (95% CI: −1.54 to 0.11) and −0.71% (95% CI: −1.54 to 0.11).

**Conclusion:**

The distribution and pattern of LGE influence the reproducibility of its quantification. FWHM provided the highest intra-observer reproducibility for sharply demarcated scars, as seen in CMI. For more diffuse fibrosis patterns, such as in HCM and IHD, both the 5-SD and 6-SD techniques offered similarly reproducible performance.

## Background

Late gadolinium enhancement (LGE) imaging is the reference standard in cardiovascular magnetic resonance (CMR) for detecting myocardial scar and fibrosis (1). A growing body of evidence supports the prognostic value of LGE extent in improving patient risk stratification and guiding therapy across various patient cohorts, including chronic myocardial infarction (CMI), hypertrophic cardiomyopathy (HCM) and inflammatory heart disease (IHD) (2–8).

Several techniques are available to quantify the left ventricular (LV) extent of LGE, including manual thresholding and semi-automated approaches such as full width at half maximum (FWHM) and signal threshold vs. reference mean, which applies a threshold of *n* standard deviations from remote normal myocardium (n-SD). These methods differ significantly in how they define pathologically hyper-enhanced voxels (9). While LGE imaging is well established for fibrosis detection, no consensus exists regarding the optimal technique for quantifying LV LGE extent in ischemic and non-ischemic cardiomyopathies (1).

In the absence of a universal reference standard, the technical performance of quantification methods can be assessed by their measurement precision (variability) (10, 11). As a key determinant of reliability, precision can be operationalized through intra-observer reproducibility, which reflects the consistency of repeated measurements by the same observer (10). Techniques which yield lower intra-observer variability are potentially more reliable for clinical and research use.

Although previous studies have evaluated inter- and intra-observer reproducibility of various LGE quantification techniques (12–14), uncertainties remain regarding the applicability across different disease entities. Available data do not clarify whether a) a single LGE quantification technique achieves the highest intra-observer reliability across different disease entities or b) different techniques perform best depending on the underlying disease.

The objective of this study was to identify the most reliable LGE quantification methods by assessing intra-observer reproducibility across three disease entities: CMI, HCM, IHD.

## Methods

### Study design and population

This study is a retrospective analysis of prospectively enrolled patient data collected between 2013 and 2022 at a single center. A total of 713 patients who had undergone short-axis LGE imaging were initially screened, from which 577 met diagnostic criteria for CMI, HCM, and IHD as outlined in the Supplementary Material. The IHD cohort comprised cases of acute and chronic myocarditis, defined according to the original and updated Lake Louise criteria (15, 16). Inclusion criteria further required the presence of a disease-typical LGE pattern and sufficient image quality as described in Supplementary Material for quantitative analysis. Cases presenting with microvascular obstruction were categorized as (sub)acute myocardial infarction and excluded from the study.

This study was conducted in accordance with the declaration of Helsinki, local legislation, and institutional requirements. Data was retrospectively acquired from prospective studies. Studies were originally approved by the local ethics committee of Charité—Universitätsmedizin Berlin and according to local legislation. Ethics approval ID and Trial registration ID of the source studies: EA2/077/10, EA1/305/14 (ISRCTN48802295), EA1/076/18, EA1/111/18 (ISRCTN16766375), EA1/087/21, EA1/088/21, EA1/198/21, EA1/042/22. Written informed consent was prospectively obtained from participants at that time.

### Image acquisition

All CMR examinations were performed on a 1.5 T scanner (AvantoFit®, Siemens Healthineers, Erlangen, Germany). LGE images were acquired 10–20 min after administration of 0.1, 0.15, or 0.2 mmol/kg of gadolinium-based contrast agent, using a 2D phase-sensitive inversion recovery (PSIR) sequence. Detailed acquisition parameters are provided in Supplementary Table S2.

### Study workflow

**Figure 1:**
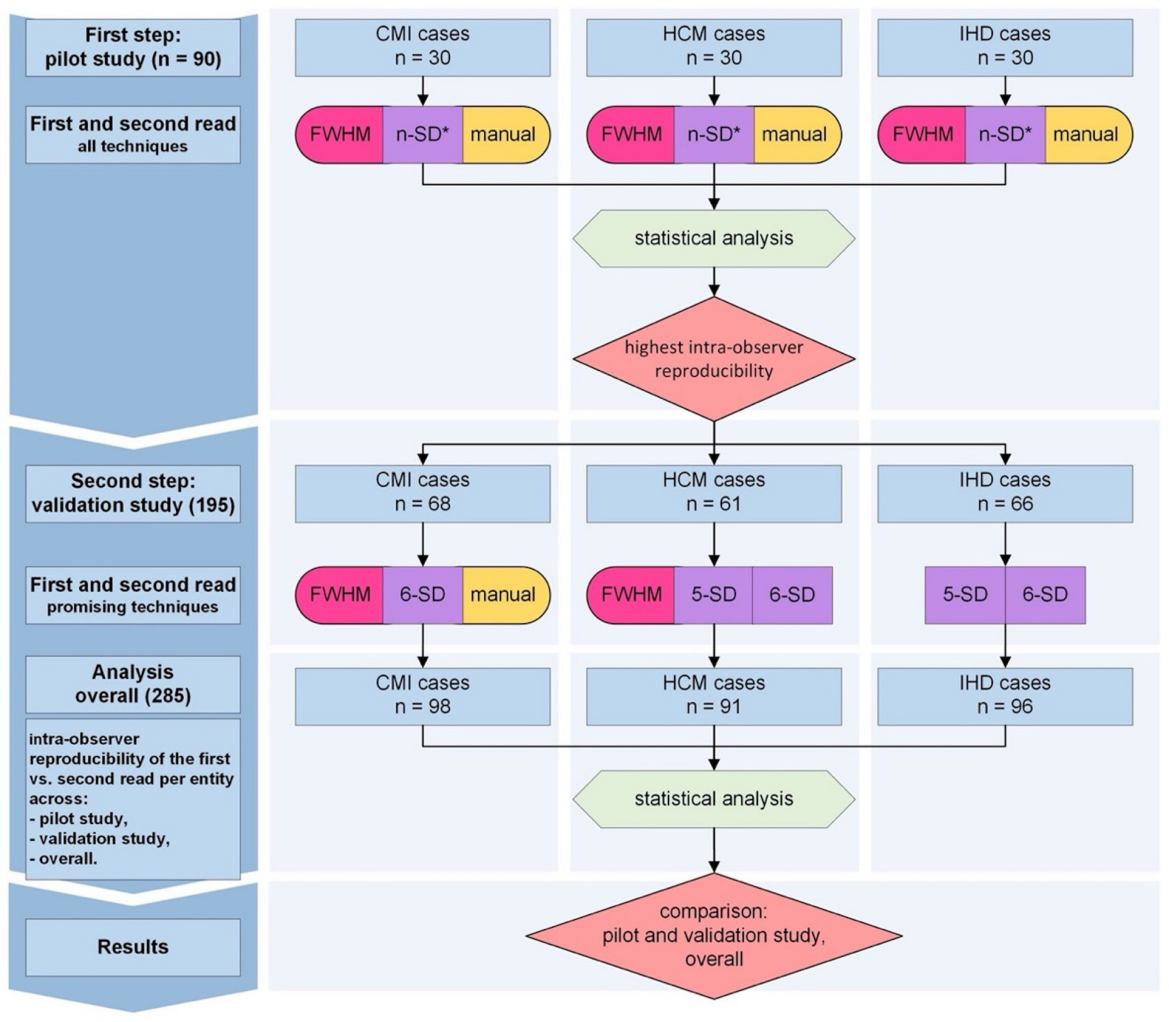
Study protocol. Intra-observer analysis followed a two-step approach. In the first step (pilot study, *n* = 90), intra-observer variability and sample size estimates were derived. LGE was quantified using seven techniques per entity: FWHM, n-SD with 2–6 standard deviations from remote normal myocardium, and manual thresholding. In the second step (validation study, *n* = 195), techniques with the lowest variability were applied in separate patient cohorts (CMI: FWHM, 6-SD, manual thresholding; HCM: FWHM, 5-SD, 6-SD; IHD: 5-SD, 6-SD). Final statistical analysis (mixed model for repeated measures) identified the techniques with the highest intra-observer reproducibility, based on results from the pilot, validation, and pooled cohorts (overall analysis). CMI, chronic myocardial infarction; HCM, hypertrophic cardiomyopathy; IHD, inflammatory heart disease; LGE, late gadolinium enhancement; FWHM, full width at half maximum; n-SD, signal threshold at *n* standard deviations from remote myocardium. 5-SD, signal threshold at 5 standard deviations from remote myocardium. 6-SD, signal threshold at 6 standard deviations from remote myocardium. *During the pilot study, all cases were analyzed using 2-, 3-, 4-, 5-, and 6-SD techniques*.*

Given the high variability in LGE quantification results reported in previous literature (10–13), a two-step study design was implemented (Figure 1). In the first step (Pilot Study), 30 cases per disease entity (CMI, HCM, IHD, *n* = 90 in total) were analyzed using all quantification techniques. Intra-observer variability was assessed to identify the techniques demonstrating the least variability within each disease group. Pilot study results subsequently informed the sample size calculation for the validation cohort.

In the second step (Validation Study, *n* = 195), only the best-performing quantification techniques from the pilot study were applied. Cases for both pilot and validation studies were randomly selected from all available eligible cases using a custom MATLAB tool (MathWorks, Natick, MA, USA), preserving proportional representation according to the size of the original source studies. LGE quantification was performed twice per case to confirm intra-observer reproducibility. In total, 759 LGE quantification analyses were completed across the two study phases.

### LGE quantification protocol

Image analysis was performed exclusively on short-axis PSIR LGE images using commercially available software (cvi42, version 5.13.7, Circle Cardiovascular Imaging, Calgary, Canada). A reader with three years of CMR experience (J.G.) conducted two separate analyses per case, blinded to previous results and separated by an interval of more than two months. A consensus read was performed with a reader with 7 years' experience (M.F.) During the pilot study, each case was analyzed using: Manual thresholding, FWHM and n-SD applying thresholds from 2 to 6 standard deviations.

### Myocardial annotation procedure

**Figure 2:**
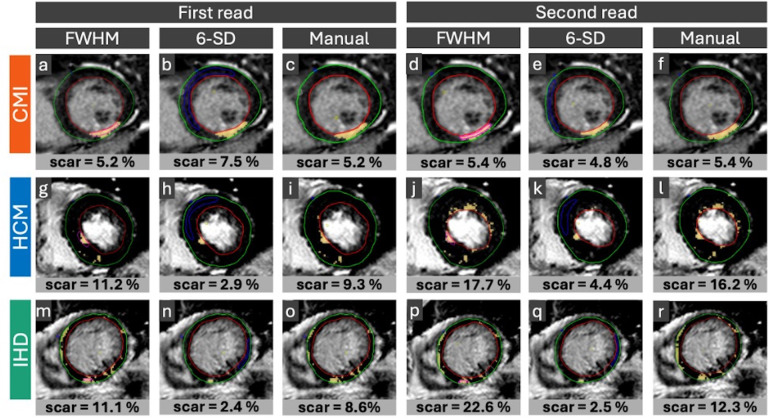
Representative LGE images and annotations. Representative cases of CMI **(a–f)**, HCM: **(g–l)** and IHD: **(m–r)** with disease-typical LGE distribution: Sharply demarcated subendocardial LGE distribution in CMI cases, patchy intramural pattern in HCM cases and subepicardial pattern in IHD cases. Horizontal rows display reader annotations in the same patient for the first and second image analysis. Vertical columns indicate LGE quantification technique applied: FWHM **(a**,**g**,**m**,**d**,**j**,**p)**, 6-SD **(b**,**h**,**n**,**e**,**k**,**q)**, and manual thresholding **(c**,**i**,**o**,**f**,**l**,**r)**. Endo- (red line) and epicardial (green line) borders were annotated. For FWHM the reference ROI (pink contour) was drawn around the visually brightest myocardial area, covering the most signal intense voxel. For the n-SD technique, the remote ROI (blue contour) was drawn in remote myocardium. Yellow marking indicates hyper-enhanced myocardium. CMI, chronic myocardial infarction; HCM, hypertrophic cardiomyopathy; IHD, inflammatory heart disease; LGE, late gadolinium enhancement; FWHM, full width at half maximum. 5-SD, signal threshold at 5 standard deviations from remote myocardium. 6-SD, signal threshold at 6 standard deviations from remote myocardium. ROI, region of interest.

Epicardial and endocardial borders were manually contoured once per read. For the FWHM technique, a region of interest (ROI) was placed around the most signal-intense voxel within the scar area on each slice (1). For the n-SD technique, a reference ROI was drawn in remote myocardium [preferably within the septum, covering two American Heart Association [AHA] (17) segments and 45% of myocardial circumference where possible] to determine 2-, 3-, 4-, 5-, and 6-SD thresholds. Manual quantification involved manually setting a threshold for each slice visually matching the extent of annotated scar areas to the area of hyperintense myocardium. Artefacts, partial volume effects, and spurious voxels (18) were manually corrected through exclusion ROIs or contour adjustments. The FWHM technique was always applied first, and myocardial contours were subsequently reused without modification for n-SD and manual thresholding analyses. Representative annotation examples are shown in Figure 2.

### Statistical analysis

In the absence of sufficient preliminary data on intra-observer variability for LGE quantification techniques, a two-step study design was implemented. A pilot study first estimated intra-observer variability in LV LGE extent, expressed as a percentage of LV mass, for each quantification method within each disease entity. These data informed sample size calculations for a subsequent validation cohort, designed to formally test intra-observer equivalence to zero within predefined clinical margins.

Sample size calculations assumed 80% power and a two-sided significance level of 5%, with equivalence defined by the 95% confidence interval (CI) for intra-observer differences lying entirely within the chosen margins. Based on pilot-derived standard deviations, a sample size of *n* = 67 patients was required for CMI (6-SD technique, SD = 2.04, margin ±2%), *n* = 89 for HCM (FWHM technique, SD = 9.44, margin ±4%), and *n* = 35 for IHD (6-SD technique, SD = 4.67, margin ±4%). A conservative sample size estimate was used to ensure sufficient power across all groups. To increase robustness, we aimed to maintain consistent group sizes in the final analysis and therefore targeted the largest required sample size (*n* ≥ 89) for all three groups.

Intra-observer reproducibility was assessed using a mixed model for repeated measures. Point estimates (PE) were calculated as the mean differences between first and second reads, with 95% CIs reported to quantify measurement precision. Bland–Altman plots were generated to visualize mean differences and 95% limits of agreement.

Results are reported separately for the pilot and validation steps and for the pooled cohort including all cases. Sample size calculations were performed using PASS 2019 (NCSS, LLC, Kaysville, Utah, USA). Statistical analyses were conducted in SAS version 9.4 (SAS Institute Inc., Cary, NC, USA). Figures were generated using GraphPad Prism version 10 (GraphPad Software, San Diego, CA), Microsoft Visio, PowerPoint version 16.79.2, and Excel version 14.7.7 (Microsoft Corporation, Redmond, WA, USA).

## Results

Out of the initially screened 713 cases, 577 cases met disease criteria, from which 464 were eligible for inclusion. Of these, 285 patients were randomly selected for the final analysis, slightly exceeding the minimum sample sizes for sufficient power in each group (CMI: *n* = 98; HCM: *n* = 91; IHD: *n* = 96). Reasons for exclusion were absence of a disease-specific LGE pattern (*n* = 136), no LGE short axis stack available (*n* = 41), poor image quality or insufficient image slices (*n* = 70), and presence of microvascular obstruction (*n* = 2). Patient characteristics are summarized in Table 1.

**Table 1:** Patient characteristics and myocardial parameters.

Parameter	Unit	CMI	HCM	IHD
Age	[years]	64.3 ± 11.4	58.2 ± 12.8	45 ± 14.1
Sex	[male/female]	73/25	62/29	61/35
BMI	[kg/m^2^]	27.6 ± 4.2	28.1 ± 4.2	26 ± 4.4
First read				
LVM	[g]	96.2 ± 28.4	137.3 ± 62.8	83.7 ± 33.6
	[ml]	91.6 ± 27.0	130.9 ± 59.8	79.8 ± 32.0
LGE extent	[g]	16.0 ± 10.1	13.0 ± 13.6	6.0 ± 7.3
	[ml]	15.2 ± 9.6	12.4 ± 13.0	6.1 ± 7.7
	[%]	16.5 ± 9.0	8.6 ± 6.8	6.4 ± 6.5
Second read				
LVM	[g]	93.0 ± 27.7	137.7 ± 60.1	81.1 ± 31.0
	[ml]	88.5 ± 26.4	131.1 ± 57.3	77.2 ± 29.5
LGE extent	[g]	14.7 ± 8.9	12.9 ± 13.6	6.1 ± 7.3
	[ml]	14.0 ± 8.5	12.3 ± 13.0	6.4 ± 7.6
	[%]	16.0 ± 8.4	8.8 ± 7.0	7.1 ± 7.2

Data are expressed as mean ± standard deviation for the overall data per disease entity. LGE extent and scarring according to FWHM in CMI and 6-SD for HCM, IHD. See supplementary material for full parameter overview. CMI, chronic myocardial infarction; HCM, hypertrophic cardiomyopathy; IHD, inflammatory heart disease; BMI, body mass index; LVM, left ventricular myocardium mass; LGE, late gadolinium enhancement; FWHM, full width at half maximum. 6-SD: signal threshold at 6 standard deviations from remote myocardium.

### CMI cases: pilot and validation studies

In the pilot study, the FWHM technique demonstrated a PE (mean ± standard error) of −0.94 ± 0.48 with a 95% CI ranging from −1.90 to 0.02, showing the most favorable intra-observer reproducibility compared to other techniques. The 6-SD technique [PE = 1.32 ± 0.48, 95% CI (0.36, 2.28)] and manual thresholding [PE = −1.47 ± 0.48, 95% CI (−2.43, −0.51)] also exhibited lower variability compared to the remaining methods, although FWHM performed best (*p* < 0.001).

**Figure 3:**
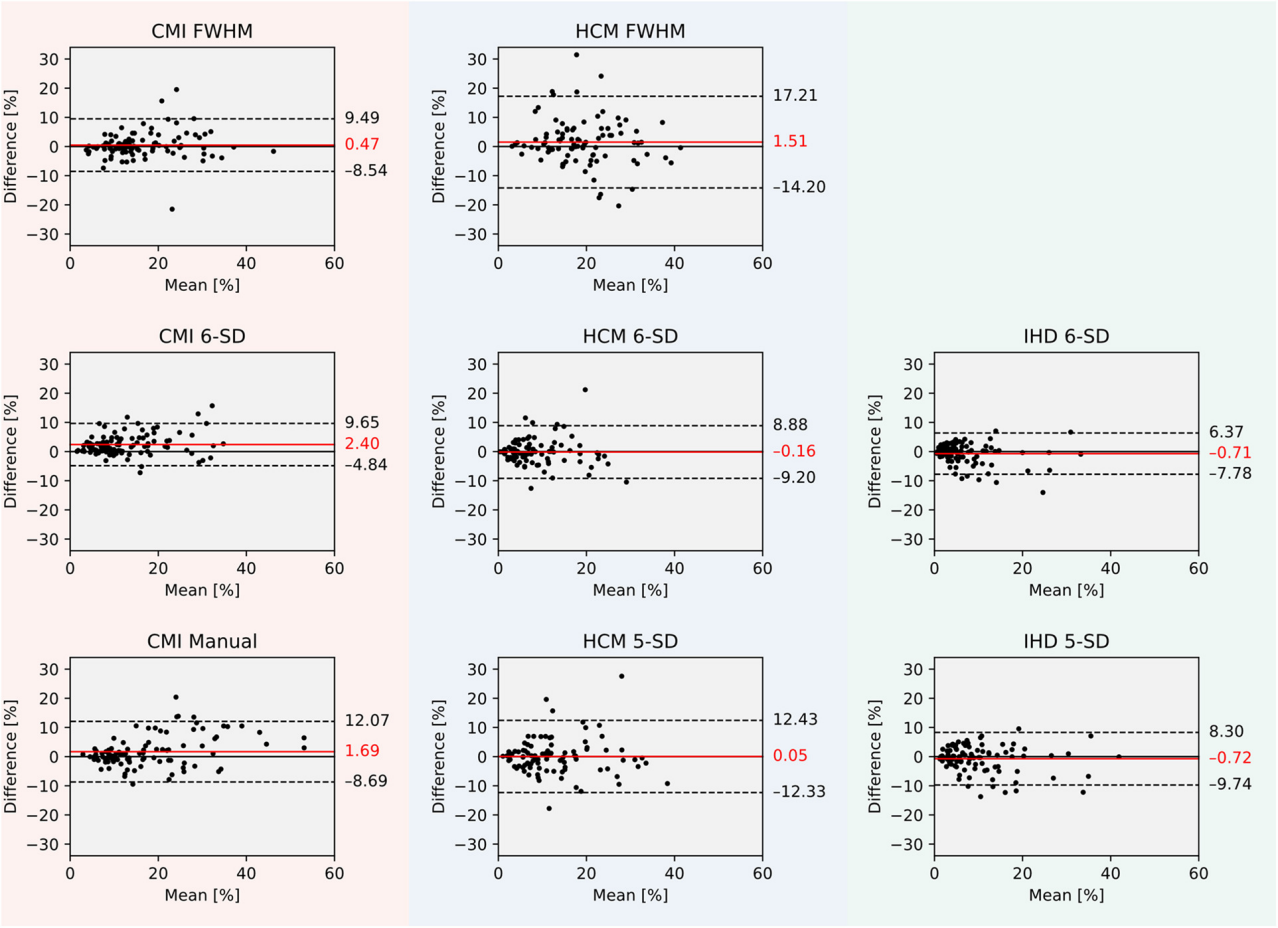
Bland–Altman plots of agreement between first and second read LGE extent in percent for all cases per quantification technique and disease entity. CMI, chronic myocardial infarction; HCM, hypertrophic cardiomyopathy; IHD, inflammatory heart disease; LGE, late gadolinium enhancement; FWHM, full width at half maximum. 5-SD, signal threshold vs. reference mean at 5 standard deviations from remote myocardium. 6-SD, signal threshold vs. reference mean at 6 standard deviations from remote myocardium.

**Figure 4:**
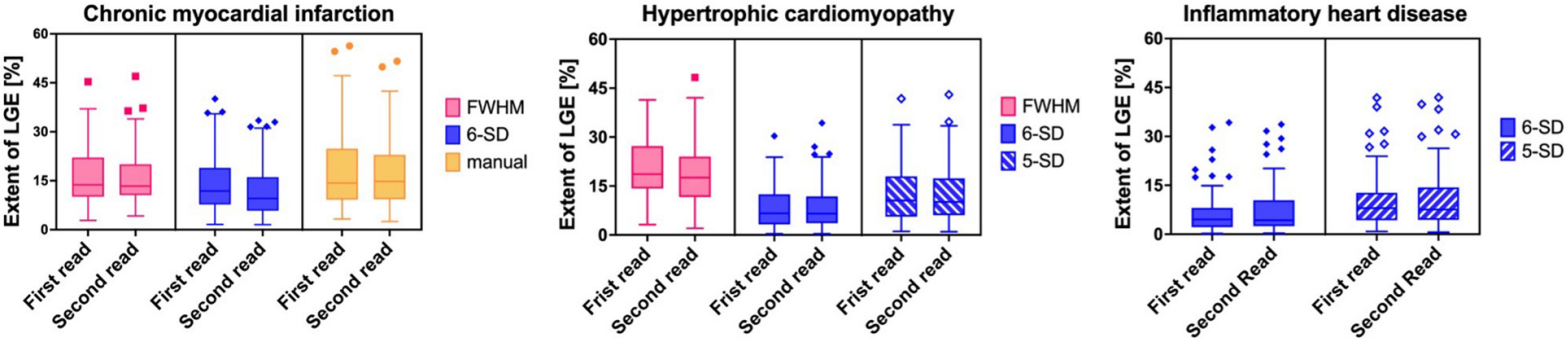
Boxplot figures of LGE extent expressed in median [%] with interquartile range (25th to 75th percentile) during first and second read per LGE quantification technique per disease entity. Whiskers depict 1.5 times interquartile range. CMI: chronic myocardial infarction. HCM, hypertrophic cardiomyopathy; IHD, inflammatory heart disease; LGE, late gadolinium enhancement; FWHM, full width at half maximum. 5-SD, signal threshold vs. reference mean at 5 standard deviations from remote myocardium. 6-SD, signal threshold vs. reference mean at 6 SD from remote myocardium.

For the validation study, FWHM, 6-SD and manual thresholding were included. FWHM again demonstrated the lowest variability [PE = 1.10 ± 0.60, 95% CI (−0.08, 2.28); *p* = 0.039]. See Figure 3 for Bland-Altman plots of agreement and Figure 4 for total LV LGE in the first and second read. Detailed results are provided in the Supplementary Material.

### HCM cases: pilot and validation studies

In the pilot study, both 5-SD [PE = −1.62 ± 1.15, 95% CI (−3.91, 0.67)] and 6-SD [PE = −1.22 ± 1.15, 95% CI (−3.51, 1.07)] techniques demonstrated lower intra-observer variability compared to other methods, including FWHM [PE = 1.49 ± 1.15, 95% CI (−0.80, 3.77)]. No significant difference was observed between 5-SD and 6-SD (*p* = 0.122).

In the validation cohort, 5-SD [PE = 0.88 ± 0.84, 95% CI (−0.77, 2.54)] and 6-SD [PE = 0.36 ± 0.84, 95% CI (−1.30, 2.01)] remained the techniques with the lowest intra-observer variability (*p* = 0.655).

### ICD cases: pilot and validation studies

In the pilot study for IHD, the 5-SD technique demonstrated a PE of −1.99 ± 0.76 with a 95% CI ranging from −3.50 to −0.47, while the 6-SD technique showed a PE of −1.31 ± 0.76 [95% CI (−2.83, 0.20)]. Both techniques exhibited considerably lower intra-observer variability compared to other methods, with no significant difference between them (*p* = 0.531).

In the validation study, reproducibility remained consistent. The 5-SD technique achieved a PE of −0.14 ± 0.50 [95% CI (−1.13, 0.85)], and the 6-SD technique a PE of −0.44 ± 0.50 [95% CI (−1.43, 0.56)]. Again, no significant difference was observed between the two methods (*p* = 0.679).

### Pooled results across all disease entities

When analyzing pooled results from both pilot and validation cohorts, the highest intra-observer reproducibility in patients with CMI was observed with the FWHM technique, which achieved a PE of 0.47 ± 0.44 and a 95% CI ranging from −0.40 to 1.35.

In HCM and IHD cohorts, both the 5-SD and 6-SD techniques demonstrated similarly high reproducibility. For HCM, the 5-SD method achieved a PE of 0.06 ± 0.68 [95% CI (−1.28, 1.39)] and the 6-SD method a PE of −0.16 ± 0.68 [95% CI (−1.50, 1.17)], with no significant difference between them (*p* = 0.251). In the IHD cohort, the 5-SD technique showed a PE of −0.72 ± 0.42 [95% CI (−1.54, 0.11)], while the 6-SD technique achieved a PE of −0.71 ± 0.42 [95% CI (−1.54, 0.11)], again without a significant difference (*p* = 0.463).

**Figure 5:**
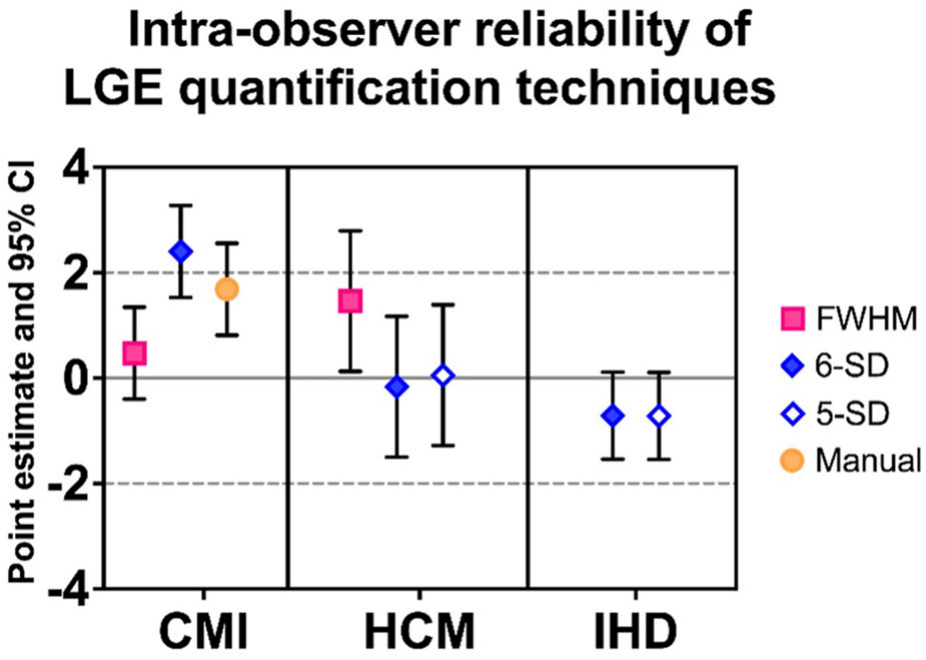
Most reliable LGE quantification techniques per entity across pilot (*n* = 90) and validation study (*n* = 195). Data are expressed as PE along with 95% CI for the overall analysis (*n* = 285). CMI, chronic myocardial infarction; HCM, hypertrophic cardiomyopathy; IHD, inflammatory heart disease; LGE, late gadolinium enhancement; PE, point estimate; CI, confidence interval; FWHM, full width at half maximum. 5-SD: signal threshold vs. reference mean at 5 standard deviations from remote myocardium. 6-SD: signal threshold vs. reference mean at 6 standard deviations from remote myocardium.

The techniques demonstrating the highest intra-observer reproducibility across all disease groups are illustrated in Figure 5**.**

### Analysis time

The average time required for LGE quantification, including manual contour tracing and application of all techniques, was as follows: Pilot study (*n* = 90): 27 min 51 s per case; Validation study (*n* = 195): 23 min 46 s per case. The most pronounced differences in analysis time were observed in HCM cases: Pilot study first read: 37 min 25 s, Pilot study second read: 25 min 57 s; Validation study first read: 23 min 21 s; Validation study second read: 19 min 57 s. Further details are provided in the Supplementary Material.

## Discussion

This single-center study assessed intra-observer reproducibility of three LGE quantification techniques (FWHM, n-SD, manual thresholding) across CMI, HCM, IHD. Our findings suggest that intra-observer reproducibility depends on scar pattern and LGE distribution. For sharply demarcated scars, as seen in CMI, FWHM achieved the highest intra-observer reproducibility. In contrast, when LGE was less well-defined, as typically observed in HCM and IHD, the 5-SD and 6-SD techniques demonstrated better reproducibility compared with FWHM.

Consistent with previous studies, FWHM had the highest reproducibility in cases with high image contrast and distinct scarring, where the most signal-intense voxel can be reliably identified (13, 19). In chronic ischemic scars, this method benefits from sharp differences in image signal intensity, allowing robust thresholding at half-maximum (13). However, in non-ischemic diseases, FWHM reproducibility decreased due to methodological challenges: less distinct scar regions, smaller differences in signal intensities between healthy and diseased myocardium, and difficulty reliably identifying the brightest voxels. Additionally, manual ROI placement and the need to exclude spurious voxels at myocardial borders contribute to higher observer dependency and time consumption.

In IHD and HCM, where fibrosis and scarring are often diffuse or patchy rather than sharply demarcated (8), n-SD techniques (5-SD and 6-SD) offered better reproducibility. Although theoretically applicable across different scar patterns, n-SD performed less reliably in CMI, likely due to difficulties in consistently positioning reference ROIs across repeated annotations. These findings align with previous studies showing n-SD's limitations for infarct quantification (18).

Our results have clinical implications. In HCM, where risk stratification thresholds such as 15% LGE extent are clinically relevant (20–22), reproducibility is critical, particularly when considering longitudinal follow-up examinations to detect meaningful increases in LGE extent, or in interventional research trials, measuring the effect of therapies over time.

Manual thresholding showed lower reproducibility across all cohorts, underlining the inherent subjectivity of human annotation (14). Even with quality assurance and systematic exclusion of artefacts, manual methods add observer bias that cannot be fully eliminated.

Interestingly, in dilated cardiomyopathy (DCM), several studies have demonstrated the prognostic value of LGE extent, most frequently quantified using the FWHM technique (23–25). A recent meta-analysis confirmed that both the presence and extent of LGE are associated with adverse outcomes, while also highlighting the need for standardized quantification protocols (26). Notably, adequately powered studies investigating intra- and inter-reader reproducibility in DCM are still lacking. In cardiac amyloidosis, LGE is primarily assessed visually based on characteristic enhancement patterns. Although transmural LGE has been shown to predict mortality (27), semi-automatic quantification is rarely used. Threshold-based techniques such as n-SD or FWHM are often not applicable due to diffuse infiltration and the absence of a clear reference myocardium.

As shown in our study, manual LGE quantification is tedious and time-consuming, hindering broader clinical application. Future work should therefore focus on automated solutions. Deep learning algorithms have demonstrated success in cine image segmentation (28, 29) and could potentially improve LGE quantification. Initiatives like the Evaluation of Myocardial Infarction from Delayed-Enhancement Cardiac MRI (EMIDEC) challenge highlight the potential of AI, although variability in the underlying manual annotations remains a hurdle (30).

### Limitations

Although this is currently the largest study examining the intra-observer reproducibility of different LGE quantification approaches, there are limitations. While our study focused on prevalent diseases with distinct LGE characteristics, uncertainties remain for diseases with other LGE patterns, such as cardiac sarcoidosis, and for cases exhibiting multiple or mixed LGE patterns within the same patient. Additional limitations of our study include the single-center and single-reader design. Further research is necessary to assess the techniques' inter-observer reproducibility and result variability across different centers with varying scanner setups and protocols.

In our study, datasets were drawn from multiple prior studies with different contrast agents, doses, and post-contrast timing. While investigating dose-, and agent-specific effects would be of scientific interest, the current study was not powered for stratified analyses by contrast dose, timing or type. Moreover, acute and chronic myocarditis may exhibit different LGE characteristics but could not be meaningfully stratified due to nonuniform scan timing in relation to disease onset in the original studies. However, both stages frequently present with similar diffuse or patchy enhancement patterns and this study focused on intra-observer reproducibility rather than pathophysiological distinction.

## Conclusions

**Figure 6:**
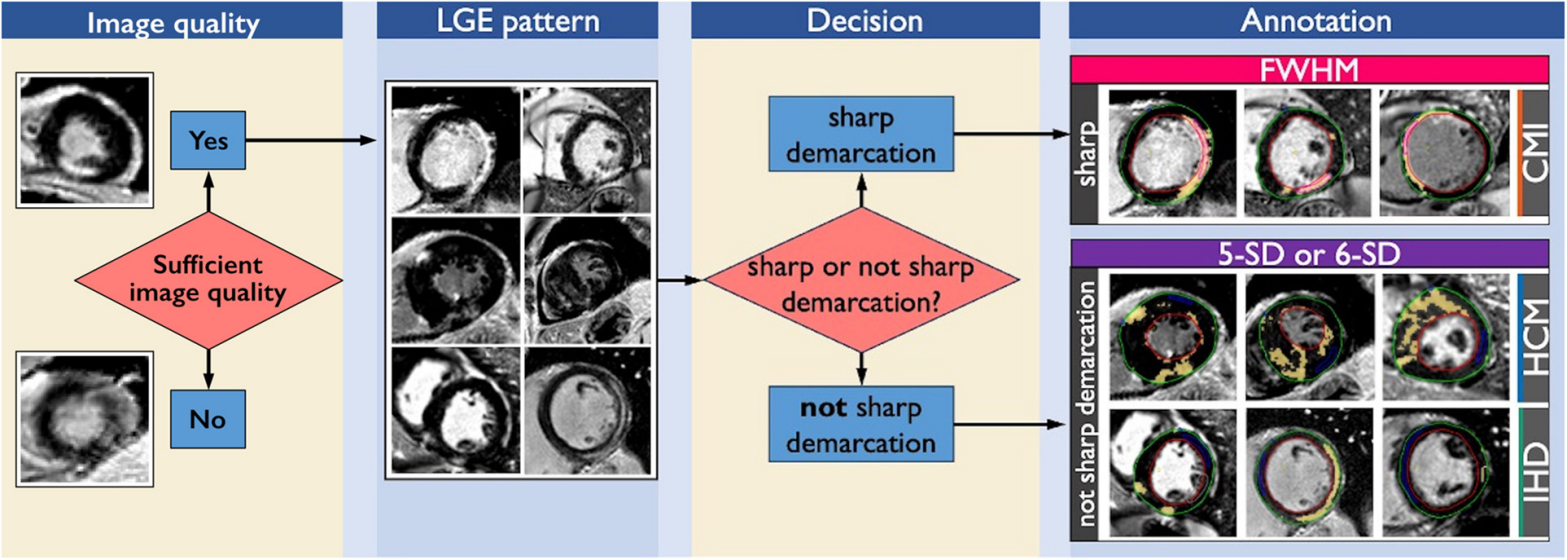
Decision tree for the first step of visual inspection. After establishing sufficient image quality, visual inspection of LGE distribution and myocardial fibrosis is the primary step. In cases with CMI, HCM and IHD LGE distribution and scar pattern can present either with sharp demarcation (CMI) or no sharp demarcation (HCM, IHD). In cases of fibrosis with sharp demarcation, that are often observed in CMI patients, FWHM is the technique with the highest intra-observer reproducibility to quantify LGE extent. In cases with focal fibrosis with not sharp demarcation as associated with HCM and IHD, 5-SD and 6-SD are equally well suited to quantify LGE extent. CMI, chronic myocardial infarction; HCM, hypertrophic cardiomyopathy; IHD, inflammatory heart disease; LGE, late gadolinium enhancement; FWHM, full width at half maximum. 5-SD: signal threshold vs. reference mean at 5 standard deviations from remote myocardium. 6-SD: signal threshold vs. reference mean at 6 standard deviations from remote myocardium.

In summary, FWHM is preferable for well-defined infarcts, while 5-SD and 6-SD techniques perform better in diffuse and/or patchy fibrosis patterns (Figure 6). Visual assessment of scar pattern should guide technique selection to maximize reproducibility in both clinical and research settings.

## Data Availability

The data analyzed in this study is subject to the following licenses/restrictions: availability of analyzed datasets is limited by German data protection laws. Patient data is not publicly available. Requests to access these datasets should be directed to Jeanette Schulz-Menger, jeanette.schulz-menger@charite.de.
