# Supplementary material for: Reproducibility of late gadolinium enhancement quantification techniques in ischemic and non-ischemic heart diseases (ReLate study)
Source: Front Cardiovasc Med. 2025 Aug 1;12:1621292. doi: 10.3389/fcvm.2025.1621292 (PMC12354482; doi:10.3389/fcvm.2025.1621292)
Supplement: Supplementary file 1 [file Datasheet1.docx]

**Supplementary Material**

|  |  |  |  |  |  |  |  |  |  |  |  |  |  |  |  |
| --- | --- | --- | --- | --- | --- | --- | --- | --- | --- | --- | --- | --- | --- | --- | --- |
| **Entity** | **Parameter** | **Unit** | **First Read** | | | | | |  | **Second Read** | | | | | |
|  |  |  |  |  |  |  |  |  |  |  |  |  |  |  |  |
|  |  |  | **FWHM** | | **Manual** | | **6-SD** | |  | **FWHM** | | **Manual** | | **6-SD** | |
| **CMI** | **LVM** | [ml] | 91.6 | ± 27.0 | 91.6 | ± 27.0 | 91.6 | ± 27.0 |  | 88.5 | ± 26.4 | 88.5 | ± 26.4 | 88.5 | ± 26.4 |
|  |  | [g] | 96.2 | ± 28.4 | 96.2 | ± 28.4 | 96.2 | ± 28.4 |  | 93.0 | ± 27.7 | 93.0 | ± 27.7 | 93.0 | ± 27.7 |
|  |  |  |  |  |  |  |  |  |  |  |  |  |  |  |  |
|  | **LGE extent** | [ml] | 15.2 | ± 9.6 | 17.4 | ± 12.9 | 13.6 | ± 9.9 |  | 14.0 | ± 8.5 | 15.1 | ± 9.8 | 10.9 | ± 8.7 |
|  |  | [g] | 16.0 | ± 10.1 | 18.3 | ± 13.6 | 14.3 | ± 10.4 |  | 14.7 | ± 8.9 | 15.8 | ± 10.3 | 11.4 | ± 9.2 |
|  |  |  |  |  |  |  |  |  |  |  |  |  |  |  |  |
|  |  | [%] | 16.5 | ± 9.0 | 18.6 | ± 12.0 | 14.1 | ± 8.7 |  | 16.0 | ± 8.4 | 16.9 | ± 10.1 | 11.7 | ± 8.0 |
|  |  |  |  |  |  |  |  |  |  |  |  |  |  |  |  |
|  |  |  | **FWHM** | | **5-SD** | | **6-SD** | |  | **FWHM** | | **5-SD** | | **6-SD** | |
| **HCM** | **LVM** | [ml] | 130.8 | ± 59.8 | 130.8 | ± 59.8 | 130.8 | ± 59.8 |  | 131.1 | ± 57.3 | 131.1 | ± 57.3 | 131.1 | ± 57.3 |
|  |  | [g] | 137.3 | ± 62.8 | 137.3 | ± 62.8 | 137.3 | ± 62.8 |  | 137.7 | ± 60.1 | 137.7 | ± 60.1 | 137.7 | ± 60.1 |
|  |  |  |  |  |  |  |  |  |  |  |  |  |  |  |  |
|  | **LGE extent** | [ml] | 26.7 | ± 18.3 | 18.4 | ± 18.1 | 12.4 | ± 13.0 |  | 24.7 | ± 19.1 | 18.0 | ± 17.1 | 12.3 | ± 13.0 |
|  |  | [g] | 28.0 | ± 19.2 | 19.4 | ± 19.0 | 13.0 | ± 13.6 |  | 25.9 | ± 20.1 | 18.9 | ± 17.9 | 12.9 | ± 13.6 |
|  |  |  |  |  |  |  |  |  |  |  |  |  |  |  |  |
|  |  | [%] | 19.8 | ± 8.8 | 13.0 | ± 9.1 | 8.6 | ± 6.8 |  | 18.3 | ± 9.7 | 12.9 | ± 9.0 | 8.8 | ± 7.0 |
|  |  |  |  |  |  |  |  |  |  |  |  |  |  |  |  |
|  |  |  |  |  | **5-SD** | | **6-SD** | |  |  |  | **5-SD** | | **6-SD** | |
| **IHD** | **LVM** | [ml] |  |  | 79.8 | ± 32.0 | 79.8 | ± 32.0 |  |  |  | 77.2 | ± 29.5 | 77.2 | ± 29.5 |
|  |  | [g] |  |  | 83.7 | ± 33.6 | 83.7 | ± 33.6 |  |  |  | 81.1 | ± 31.0 | 81.1 | ± 31.0 |
|  |  |  |  |  |  |  |  |  |  |  |  |  |  |  |  |
|  | **LGE extent** | [g] |  |  | 8.6 | ± 9.4 | 5.8 | ± 7.3 |  |  |  | 8.8 | ± 9.0 | 6.1 | ± 7.3 |
|  |  |  |  |  |  |  |  |  |  |  |  |  |  |  |  |
|  |  | [ml] |  |  | 9.1 | ± 9.9 | 6.1 | ± 7.7 |  |  |  | 9.2 | ± 9.5 | 6.4 | ± 7.6 |
|  |  | [%] |  |  | 9.9 | ± 8.1 | 6.4 | ± 6.5 |  |  |  | 10.5 | ± 8.8 | 7.1 | ± 7.2 |

***Table 1:*** *Results of quantification in first and second read per disease entity. Data are expressed as mean with one standard deviation FOR LVM in (ml) and (g) and LGE extent in (ml), (g), (%). CMI: Chronic myocardial ischemia. BMI: Body mass index. HCM: Hypertrophic cardiomyopathy. IHD: inflammatory heart disease. LGE: Late gadolinium enhancement. LVM: Left ventricular myocardium mass.*

| **CMI** | **HCM** | **IHD** | **total** | **scans acquired** | **time after GBCA** | **GBCA dosage** | **GBCA** | **Recon** | **Sequence** | **TI, TR, TE, FA** | **FOV** | **matrix** | **voxel size** | **ST** | **slices gap** |
| --- | --- | --- | --- | --- | --- | --- | --- | --- | --- | --- | --- | --- | --- | --- | --- |
|  |  | 13 | 13 | September 2013 - May 2015 | 15 min | 0.15 mmol/kg | gadobutrol (Gadovist®, Gadovist; Bayer Healthcare, Germany) | PSIR | LGE fast sequences | TI 300ms, TR 10.4 ms, TE 5.4ms, FA 30° | 350x263 mm2 | 256x197 mm | 1.4x1.4 mm2 | 7mm | 0 mm |
| 25 |  |  | 25 | August 2018 - June 2019 | 15 min | 0.2 mmol/kg | gadoteridol (ProHance®, Bracco S.p.A., Milan, Italy) | PSIR | SSFP sequences | TI 240 ms, TR 29.76 ms, TE 5.17 ms, FA 30° | 350x263 mm2 | 256x192 mm | 1.4x1.4 mm2 | 7 mm | 0 mm |
| 14 |  | 16 | 30 | August 2018 - December 2020 | 15 min | 0.2 mmol/kg | gadoteridol (ProHance®, Bracco S.p.A., Milan, Italy) | PSIR | SSFP sequences | TI 300ms, TR 10.2 ms, TE 2.5 ms, FA 30° | 350x450 mm2 | 256x192 mm | 1.4x1.4 mm2 | 7 mm | 0 mm |
| 2 |  | 31 | 33 | August 2020 - November 2021 | 10-20 min | 0.2 mmol/kg | gadoteridol (ProHance®, Bracco S.p.A., Milan, Italy) | PSIR | gradient echo sequences | TI 240 ms, TR 29.76 ms, TE 5.17 ms, FA 30° | 350x263 mm2 | 256x166 mm | 1.4x1.4 mm2 | 7 mm | 0 mm |
|  | 25 |  | 25 | July 2015 - October 2018 | 15 min | 0.2 mmol/kg | gadoteridol (ProHance®, Bracco S.p.A., Milan, Italy) | PSIR | SSFP sequences | TI 260 ms, TR = 7.70 ms, TE 5.4 ms, FA 30° | 350x263 mm2 | 192x125 mm or 192x174 mm | 1.4x1.4 mm2 | 8 mm | 0 mm |
|  | 31 |  | 31 | October 2015 - Dezember 2021 | 10-20 min | 0.2 mmol/kg | gadoteridol (ProHance®, Bracco S.p.A., Milan, Italy) | PSIR | SSFP sequences | TI 240 ms, TR 29.76 ms, TE 5.17 ms, FA 30° | 350x263 mm2 | 192x125 mm or 192x174 mm or 192x256 mm | 1.4x1.4 mm2 | 7 mm or 8 mm | 0 mm |
| 57 |  |  | 57 | October 2014 - February 2016 | 10-20 min | 0.2 mmol/kg | CMI: gadoteridol (ProHance®, Bracco S.p.A., Milan, Italy) | PSIR | multi-slice 2D bSSFP-based | TI 240ms, TR 29.76ms, TE 5.17ms, FA 65° | 350x450 mm2 | 144x192 mm | 1.4x1.4 mm2 | 7 mm | 0 mm |
|  | 35 | 36 | 71 | November 2014 - July 2015 | 10-20 min | 0.1 mmol/kg or 0.15 mmol/kg | IHD: gadopentetate (Magnevist®, Bayer Healthcare, Wayne, New Jersey, USA) | PSIR | multi-slice 2D bSSFP-based | TI 240ms, TR 29.76ms, TE 5.17ms, FA 65° | 350x450 mm2 | 144x192 mm | 1.4x1.4 mm2 | 7 mm | 0 mm |
| 98 | 91 | 96 | 285 |  |  |  |  |  |  |  |  |  |  |  |  |
|  |  |  |  |  |  |  |  |  |  |  |  |  |  |  |  |
| ***Table 2: Image acquisition parameters.*** *bSSFP: balanced steady state free precession; CMI: Chronic myocardial infarction; FA: Flip angle; FOV: Field of view; GBCA: gadolinium based contrast agent; HCM: Hypertrophic cardiomyopathy; IHD: Inflammatory heart disease; LGE: Late gadolinium enhancement; PSIR: phase sensitive inversion recovery****;*** *Recon: Reconstruction; ST: Slice thickness; SSFP: Steady state free precession; TE: Echo Time TI: Inversion time; TR: Repetition Time.* | | | | | | | | | | | | | | | |

|  |  |  |  |  |  |  |
| --- | --- | --- | --- | --- | --- | --- |
|  | **pilot study** | | **validation study** | | **all cases** | |
| **cohort** | **1st read** | **2nd read** | **1st read** | **2nd read** | **pilot** | **validation** |
| **CMI** | 28:50 | 24:46 | 23:21 | 23:30 | 26:48 | 23:25 |
| **HCM** | 37:25 | 25:57 | 23:21 | 19:57 | 31:41 | 21:39 |
| **IHD** | 26:39 | 23:30 | 27:52 | 24:37 | 25:04 | 26:15 |
| **all** | 30:58 | 24:44 | 24:51 | 22:41 | 27:51 | 23:46 |
| ***Table 3:*** *Contouring time expressed in minutes and seconds for manual tracing and applying quantification techniques that were observed in the validation study (mm:ss). CMI: Chronic myocardial infarction; HCM: Hypertrophic cardiomyopathy; IHD: Inflammatory heart disease.* | | | | | | |
|  |  |  |  |  |  |  |

**Diagnostic Inclusion Criteria**

For inflammatory heart disease (IHD), diagnosis was based on the original or updated Lake Louise Criteria (1,2), depending on the date of the initial scan. A diagnosis of acute IHD required the appropriate clinical context plus:

1. LGE consistent with myocardial inflammation and regional injury and
2. either T2-weighted imaging showing a myocardium-to-skeletal muscle ratio >1.9 or elevated segmental T2 values on T2 mapping or
3. myocardial hyperemia or capillary leakage, defined as a >70% increase in myocardial signal intensity between pre- and early post-gadolinium images reflecting a higher specificity than the published cut-off of 45% (original Lake Louise Criteria)

A diagnosis of chronic IHD was made if LGE showed an intramural or subepicardial pattern consistent with scar and fibrosis, in the absence of oedema on T2-weighted imaging or mapping.

Chronic myocardial infarction (CMI) was diagnosed in the appropriate clinical context and if LGE imaging demonstrated a typical subendocardial or transmural enhancement pattern consistent with ischaemic scar, and no evidence of oedema on T2-weighted imaging or mapping (3). Cases with microvascular obstruction (MVO) were excluded.

Hypertrophic cardiomyopathy (HCM) was diagnosed based on imaging evidence of left ventricular hypertrophy (maximum wall thickness ≥15 mm at any site) in the absence of other cardiac, metabolic, or systemic conditions capable of producing similar hypertrophy (4).

**Image Quality Criteria**

Image quality was considered suitable for LGE quantification if the following criteria were met:

1. clear delineation of endocardial and epicardial borders,
2. fibrosis or scar visibly distinguishable from surrounding tissue,
3. both the scar region and reference (remote) myocardium free of relevant artifacts, and
4. minor artefacts affecting less than 25% of a myocardial segment, and not overlapping with areas of fibrosis, were accepted.

Regions affected by artifacts were manually excluded from analysis.

**References**

1. Ferreira VM, Schulz-Menger J, Holmvang G, Kramer CM, Carbone I, Sechtem U, Kindermann I, Gutberlet M, Cooper LT, Liu P, et al. Cardiovascular Magnetic Resonance in Nonischemic Myocardial Inflammation. *J Am Coll Cardiol* (2018) 72:3158–3176. doi: 10.1016/j.jacc.2018.09.072

2. Friedrich MG, Sechtem U, Schulz-Menger J, Holmvang G, Alakija P, Cooper LT, White JA, Abdel-Aty H, Gutberlet M, Prasad S, et al. Cardiovascular Magnetic Resonance in Myocarditis: A JACC White Paper. *J Am Coll Cardiol* (2009) 53:1475–1487. doi: 10.1016/j.jacc.2009.02.007

3. Abdel-Aty H, Zagrosek A, Schulz-Menger J, Taylor AJ, Messroghli D, Kumar A, Gross M, Dietz R, Friedrich MG. Delayed Enhancement and T2-Weighted Cardiovascular Magnetic Resonance Imaging Differentiate Acute From Chronic Myocardial Infarction. *Circulation* (2004) 109:2411–2416. doi: 10.1161/01.CIR.0000127428.10985.C6

4. Maron BJ, Desai MY, Nishimura RA, Spirito P, Rakowski H, Towbin JA, Rowin EJ, Maron MS, Sherrid MV. Diagnosis and Evaluation of Hypertrophic Cardiomyopathy. *J Am Coll Cardiol* (2022) 79:372–389. doi: 10.1016/j.jacc.2021.12.002
